# A Coalitional Formation Game for Physical Layer Security of Cooperative Compressive Sensing Multi-Relay Networks

**DOI:** 10.3390/s18092942

**Published:** 2018-09-04

**Authors:** Jialun Li, Shuai Chang, Xiaomei Fu, Liang Zhang, Yishan Su, Zhigang Jin

**Affiliations:** 1 School of Marine Science and Technology, Tianjin University, Tianjin 300072, China; Li_five@tju.edu.cn (J.L.); liangzhang@tju.edu.cn (L.Z.); 2 School of Electrical and Information Engineering, Tianjin University, Tianjin 300072, China; yishan.su@tju.edu.cn (Y.S.); zgjin@tju.edu.cn (Z.J.)

**Keywords:** coalitional game, physical layer security, cooperative compressive sensing, multi-relay networks

## Abstract

Cooperative relaying is an effective technology to improve the capacity of physical-layer security, in which the relay helps forward the received signal to the destination. In this paper, a cooperative compressive sensing and amplify-and-forward (CCS-AF) scheme, which combines the compressive sensing theory and amplify-and-forward strategy, is proposed to increase the secrecy capacity. To optimize the secrecy performance, a coalition formation algorithm based on coalitional game theory of optimal relay selection is proposed to maximize the secrecy capacity. Different to maximizing the individual utility based on the traditional pareto order, the max-coalition order rule is newly defined to guide the coalitional formation. Simulation results indicate that with the proposed algorithm, part of the relays could form a coalition to forward the information and the proposed algorithm could significantly improve the secrecy capacity of cooperative multi-relay networks.

## 1. Introduction

Security problems have always been of great importance in wireless communication. Traditionally, the transmitted data is encrypted by the keys to assurance the security. However, the commonly used cryptographic mechanisms have several main shortcomings, such as the difficulty in distributing the key over a public medium and the high computational complexity in wireless sensor networks (WSNs).

Recently, physical layer security has attracted much attention. It could enhance the security of wireless communication against eavesdroppers by exploiting the physical characteristics of wireless channels without encryption keys [1]. The definition of secrecy capacity is the maximum rate of secret information sent from the user to its destination in the presence of eavesdroppers [2,3,4]. It equals to the channel capacity difference between the main channel and the eavesdropper channel. However, to achieve the security, the channel condition limitation must be satisfied for networks without relays. The limitation is that the security can be achieved only when the main channel (source to–relay channel) is better than the eavesdropper channel (source to eavesdropper channel) [5]. To break through the limitation, the cooperative relay, which helps to improve the main channel condition, has recently been used as a practical technology to provide secret transmission [3,4,5,6,7].

Many kinds of approaches have been proposed in light of the physical-layer security under the cooperative communication framework, such as relay strategies and relay selection schemes. For relay strategies, amplify-and-forward (AF) and decode-and-forward (DF) are the most commonly used strategies [8,9,10,11]. For relay selection schemes, single-relay selection (SRS) and multi-relay selection (MRS) schemes are proposed to improve the secrecy capacity based on the signal-to-noise ratio (SNR) at the destination [12,13,14]. These relay selection schemes all require a centralized controller and are not suitable for the practical networks.

The characteristic of self-organization is of great importance to the distributed WSNs and cannot be realized by the traditional MRS schemes. Thus, game theory has attracted much attention in WSNs. Game theory, a highly appealing mathematical tool, has been used to design algorithms for cooperation and competition scenarios in wireless networks. It has also been applied to the physical-layer security for relay selection and power optimization [15,16,17,18,19,20,21,22,23,24]. In relay selection schemes, the distributed coalitional game has been studied in wireless networks, where users can self-organize into disjointed coalitions and maximize the secrecy capacity of each user [15,16,17]. The work in [18,19] solves the problem of multi-relay selection via a distributed algorithm based on the political coalition formation game and offers a network sum-rate performance/stability tradeoff. Furthermore, in power optimization, an efficient power-allocation protocol based on the coalition formation scheme is proposed in [20]. It achieves a significant reduction in power consumption and an improvement in payoff for transmitter nodes. A distributed coalition formation algorithm applied to a cognitive radio (CR) network utilizes the power of secondary users to interfere with the eavesdroppers in order to improve the primary user’s secrecy capacity [21]. In [22], a two-level decentralized approach based on game theory is proposed to solve the relay selection and resource allocation problems of the D2D networks. In this approach, D2D pairs and relays can form and swap coalitions, and the system capacity can be enhanced significantly. The nontransferable coalition formation game has been employed in [23] for relay selection in cooperative networks with energy harvesting relays, and it can approach the empirical bounds of the capacity very closely. Furthermore, coalition game theory is also utilized in [24] to select the optimal relay pairs for improving the secure key generation rate in D2D communications with nontrusted relays.

Compressive sensing (CS) has recently been widely used in wireless communication and signal processing. The CS could capture and represent the compressible signals at a rate far below the Nyquist rate, and the information could be retrieved from a small number of linear measurements [25,26,27]. The CS method has been studied as a physical layer security strategy of wireless networks in many research works. In [28,29], CS is used in a wireless network with one source node, one receiver, and one eavesdropper, and the measurement matrix is used as an encryption key to achieve the computational secrecy. Furthermore, authors in [30,31] considered the keyless PHY-layer secrecy scenario. The measurement matrix in [31] is the encryption key to encode the source’s signal, and the secrecy is ensured. Those studies indicate that the measurement matrix can be used to achieve the physical-layer security without the channel state information of eavesdroppers.

However, the networks of the above studies are based on the point-to-point (one source and one destination) communication scenario, and due to the decentralized property of the WSNs, the CS methods in [28,29,30,31] are not directly applicable to the WSNs [32]. In [33], authors firstly utilize the CS-AF (compressed sensing amplify and forward) scheme in WSNs. It indicates that the channel matrix from sources to relays can be used as the secure measurement matrix to achieve security. The results show that the probability of recovery of the eavesdroppers under the CS-AF scheme can be arbitrarily small; or equivalently, in terms of physical security, the CS-AF scheme can dramatically enhance the protection against eavesdropper. However, the influences of relays are not considered.

The main contributions are as follows:

(1) We propose a CCS-AF (cooperative compressed sensing amplify and forward) scheme for physical layer security in wireless sensor network, in which relays amplify and forward the received combined sources’ signals. We find that secrecy capacity is not proportional to the number of relays and the secrecy capacity is also sensitive to the source–relay distances.
(2) The max-coalition order is newly defined to guide the coalition formation, which is different from the traditional ‘Pareto order’. It is used to optimize the whole network utility instead of the individual utility.
(3) A coalitional formation algorithm for multi-relay selection is proposed to find the optimal coalition structure to achieve the maximum secrecy capacity. The simulation results show that the proposed algorithm can improve the secrecy capacity compared with the circumstance with all relays. Furthermore, the distributed algorithm confers to the relays self-organization and self-optimization properties.

The rest of this paper is organized as follows: In Section 2, a cooperative network model with multi-user and multi-relay is proposed based on CCS-AF. A brief introduction of coalitional game is shown in Section 3, and the coalition formation algorithm is introduced in Section 4. The results of simulations are provided and analyzed in Section 5. Finally, conclusions are drawn in Section 6.

## 2. System Model

**Figure 1:**
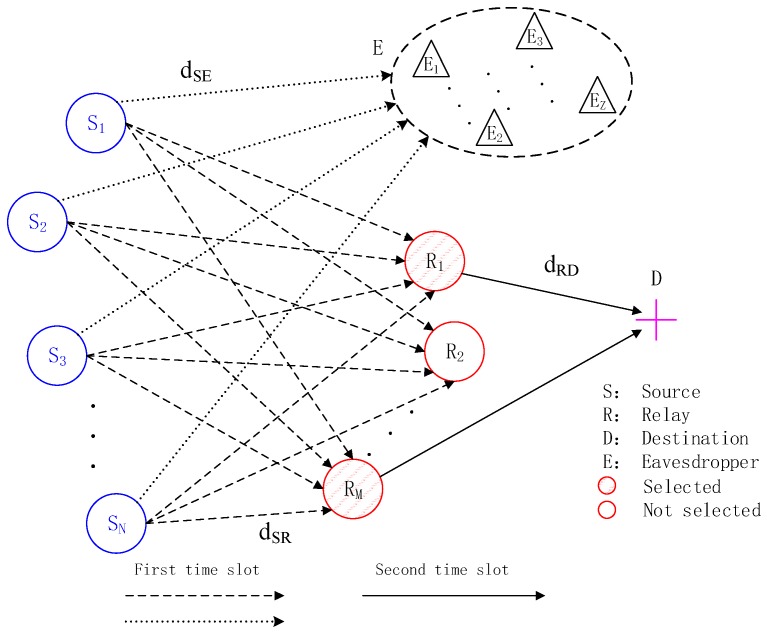
CCS-AF model with eavesdroppers.

In this section, we model a cooperative system with multiple sources and multiple relays based on CS. As shown in Figure 1, there are N sources ($S_{1},\ldots ,S_{N}$), M relays ($R_{1},\ldots ,R_{M}$), Z eavesdroppers ($E_{1},\ldots ,E_{Z}$), and one destination (D; M<N) in the network. The transmission period is divided into two time slots. In the first time slot, the source nodes send their own information to the relay nodes simultaneously. In the second time slot, some or all of the relays will be selected to amplify and forward (AF) the received combined signals to the destination. Besides, the channel state information (CSI) is known to all legitimate users.

The sparsity of the sources’ signal is denoted as K (K≪N) [34]. Let $H_{SR}\in R^{M\times N}$ denote the source–relay channel matrix, the matrix $H_{DR}\in R^{M\times 1}$ denote the relay–destination matrix vector, and the matrix $H_{SE}\in R^{Z\times M}$ denote the source–eavesdropper channel matrix. The source–relay distances, relay–destination distances, and source–eavesdropper distances are represented as $d_{SR}$, $d_{RD}$, and $d_{SE}$, respectively. Secrecy capacity, which is the channel capacity difference between the main channel and the eavesdropper channel, is used to measure the system security. It can be represented as

(1) $$C_{S}={\left(C_{D}-C_{E}\right)}^{+}$$

where $C_{S}$ is the secrecy capacity, $C_{D}$ is the capacity at destination, and $C_{E}$ is the capacity at the eavesdropper.

### 2.1. CCS-AF Scheme

In the CCS-AF scheme, there are there phases, namely the sensing phase, measurement phase, and reconstruction phase.

#### 2.1.1. Sensing Phase

In the sensing phase, only *K* of *N* source nodes whose readings are changed in a certain quantity will transmit their data to the relay nodes synchronously. Let $x_{i}$ denote the signal transmitted by the source $S_{i}$. Thus, there are *K* nonzero elements and *N* − *K* zero elements in the *N*-dimensional sources’ signal $X=\left[x_{1},x_{2},\ldots ,x_{N}\right]$. According to the CS theory, the number of nonzero elements in the signal is called the sparsity. Hence, the sparsity of sources’ signal X is *K*.

#### 2.1.2. Measurement Phase

In this CCS-AF scheme, the transmission matrix is used as the measurement matrix of CS. Modeled by the source–relay transmission matrix, the signal received at each relay is the linear combination of the sources’ signal X. Furthermore, the X signal is compressed from the *N* dimension to the *M* dimension through the M × N source–relay transmission matrix.

According to the CS theory, the measurement should satisfy the restricted isometry property (RIP). The transmission matrix is proved as the measurement matrix in the following part.

In the first time slot, the sources send information to relays simultaneously. The power constraints of the sources and relays are $P_{S}$ and $P_{R}$, respectively. The source–relay channel matrix $H_{SR}\sim N\left(0,M^{-1}\right)$ is incoherent with the identity matrix, and satisfies the RIP property with a high probability as long as M≥c·Klog(N/K), where M is the number of relays, c is a small constant, K is the sparsity, and N is the number of sources [33]. In this paper, the number of relays M should satisfy Equation (2) [29,34]:

(2) 2K≤M<N

The transmission matrix $A\in R^{M\times N}$ is equal to $A=\alpha_{SR}H_{SR}$. Besides, $\alpha_{SR}$ is a M×N matrix which represents the path loss of the first time slot, and the path loss from the jth source ($S_{j}$) to the ith relay ($R_{j}$) is $\alpha_{SR}^{i,j}=d_{i,j}^{-\mu /2}\left(i\in \left[1,M\right];j\in \left[1,N\right]\right)$. The received signal of relays is represented as

(3) $$Y_{R}=A\cdot X+N_{0}$$

where $N_{0}\in R^{M\times 1}$ is an additive white Gaussian noise (AWGN) vector at relays and the variance is $\sigma_{n_{0}}^{2}$.

In the second time slot, the selected relays amplify and forward the received sources’ signal to the destination (D). The diagonal matrix $H_{RD}\in R^{M\times M}$ represents the channel between relays and the destination. The relay–destination transmission matrix is $H=\alpha_{RD}H_{RD}$, where $\alpha_{RD}\in R^{M}$ is the relay–destination path loss. Thereby, the received signal at the destination is

(4) $$Y_{D}=\beta \cdot H\cdot Y_{R}+W_{0}=\beta \cdot H\cdot \left(A\cdot X+N_{0}\right)+W_{0}$$

where β is the diagonal matrix and its entity $\beta_{ii}=\sqrt{\frac{P_{R}/M}{\frac{P_{S}}{N}\cdot \sum_{j=1}^{N}{\left|A_{i,j}\right|}^{2}+\sigma_{n_{0}}^{2}}}\left(i\in \left[1,M\right];j\in \left[1,N\right]\right)$ denotes the amplification coefficient of $R_{i}$, and $W_{0}\in R^{M\times 1}$ is the noise between relays and D and is a AWGN vector with variance $\sigma_{w_{0}}^{2}$.

Equation (4) could be rewritten as

(5) $$Y_{D}=\Phi \cdot X+\left(\beta \cdot H\cdot N_{0}+W_{0}\right)$$

where Φ=β·H·A. The Φ matches the requirement of the RIP property, because the transmission matrix $A\in R^{M\times N}$ satisfies the RIP property and β·H is a diagonal matrix. Therefore, the transmission matrix Φ can be used as the secure measurement matrix to encrypt the sources’ signal X.

#### 2.1.3. Reconstruction Phase

The destination receives all the signals from the relays and can recover the sources’ signal X from $Y_{D}$ by solving the convex optimization problem:

(6) $$\min{||\hat{X}||}_{l_{1}}\;s.t.\;{||Y_{D}-\Phi \hat{X}||}_{l_{2}\;}\leq \varepsilon$$

where ε is an upper bound on the magnitude of the noise and $\hat{X}$ is the reconstructed vector at *D*.

It should be noted that the eavesdroppers in this model are unable to reconstruct the original signal because they have no knowledge about the measurement matrix. Thus, the eavesdropper cannot overhear the signal from the relay nodes, and the security performance is improved.

### 2.2. Secrecy Capacity

To calculate secrecy capacity conveniently, we use the singular value decomposition (SVD) method introduced in [35,36] to obtain the equivalent parallel channel. The M × N source–relay channel matrix A is transformed to a M×1 parallel channel vector.

Let $A=U\Lambda V^{H}$, and then the signal at the relays can be represented by $Y_{R}=U\Lambda V^{H}\cdot X+N_{0}$, where Λ is a diagonal matrix, $U\in C^{M\times M}$ and $V\in C^{M\times N}$ are both unitary matrixes, and $V^{H}$ is the conjugate transpose of V. Hence, the signal received at the relays shown in Equation (3) can be transformed as follows:

(7a) $$Y_{R}=U\Lambda V^{H}\cdot X+N_{0}$$

(7b) $$U^{H}Y_{R}=U^{H}U\Lambda V^{H}\cdot X+U^{H}N_{0}\;$$

(7c) $$Y_{R}^{\prime }=\Lambda X^{\prime }+N_{0}^{\prime }$$

As is shown, in Equation (7c), $Y_{R}^{\prime }=U^{H}\cdot Y_{R}$, $X^{\prime }=V^{H}\cdot X$, and $N_{0}^{\prime }=U^{H}\cdot N_{0}$. Then, bringing Equation (7c) into Equation (4), the received signal at the destination can be written as

(8) $$Y_{D}^{\prime }=\beta \cdot H\cdot Y_{R}^{\prime }+W_{0}\;\;=\beta \cdot H\cdot \Lambda \cdot X+\beta \cdot H\cdot N_{0}^{\prime }+W_{0}\;$$

Due to the property of the unitary matrix, the power of signals $Y_{R}$,X, and $N_{0}$ cannot be changed by the U and V, and we can know that $P_{X^{\prime }}=P_{X}$, $P_{N_{0}^{\prime }}=P_{N_{0}}$, and $P_{Y_{R}^{\prime }}=P_{Y_{R}}$. Thus, the signal power and the noise power could be formulated as follows:

(9a) $$P_{i}=\frac{P_{S}}{N}\cdot {\left|H_{ii}\right|}^{2}\beta_{ii}^{2}\Lambda_{i}^{2}$$

(9b) $$\sigma_{i}^{2}=\sigma_{w_{0}}^{2}+\sigma_{n_{0}}^{2}{\left|H_{ii}\right|}^{2}\beta_{ii}^{2}$$

where i indicates the ith parallel Gaussian channel and $\Lambda_{i}$ is the ith diagonal entry of Λ.

Then, the channel capacity C of the main channel is

(10) $$\begin{array}{ll} C & =\frac{1}{2}\overset{M}{\underset{i=1}{\sum }}\log_{2}\left(1+P_{i}/\sigma_{i}^{2}\right)=\frac{1}{2}\overset{M}{\underset{i=1}{\sum }}\log_{2}\left(1+\frac{\frac{P_{S}}{N}\cdot {\left|H_{ii}\right|}^{2}\Lambda_{i}^{2}}{\frac{\sigma_{w_{0}}^{2}}{\beta_{ii}^{2}}+\sigma_{n_{0}}^{2}{\left|H_{ii}\right|}^{2}}\right)\\ & =\frac{1}{2}\overset{M}{\underset{i=1}{\sum }}\log_{2}\left(1+\frac{\frac{P_{S}}{N}\cdot {\left|H_{ii}\right|}^{2}\Lambda_{i}^{2}}{\frac{\sigma_{w_{0}}^{2}}{P_{R}/M}\left(\frac{P_{S}}{N}\cdot \sum_{j=1}^{N}{\left|A_{ij}\right|}^{2}+\sigma_{n_{0}}^{2}\right)+\sigma_{n_{0}}^{2}{\left|H_{ii}\right|}^{2}}\right) \end{array}$$

where $\sigma_{n_{0}}^{2}$ and $\sigma_{w_{0}}^{2}$ are the noise powers of the two time slots, respectively.

The signal received by the eavesdropper $E_{f}$ could be expressed as

(11) $$Y_{E_{f}}=B_{f}X+N_{E_{f}}$$

where $B_{f}$ is the transmission vector between the sources and eavesdropper $E_{f}$ and $N_{E_{f}}$ is the noise at $E_{f}$. The power of the signal and the noise at $E_{f}$ are $P_{f}^{e}=\frac{P_{S}}{N}\cdot {\left|B_{j,f}\right|}^{2}$ and $\sigma_{E_{f}}^{2}$, respectively. The channel capacity from $S_{j}$ to $E_{f}$ is

(12) $${\hat{C}}_{j,f}^{e}=\frac{1}{2}\log_{2}\left(1+\frac{\frac{P_{S}}{N}\cdot {\left|B_{j,f}\right|}^{2}}{\sigma_{E_{f}}^{2}}\right)$$

where $B_{j,f}$ is the path loss between $S_{j}$ and $E_{f}$, and $\sigma_{E_{f}}^{2}$ is the noise at $E_{f}$.

Therefore, the eavesdropper channel capacity is as follows:

(13) $$C_{E}=\overset{N}{\underset{j=1}{\sum }}\max\;\left({\hat{C}}_{j,1}^{e},\;\ldots ,{\hat{C}}_{j,t}^{e},\ldots ,{\hat{C}}_{j,Z}^{e}\right)$$

The secrecy capacity of the CCS-AF system can be represented as $C_{S}=C-C_{E}$ based on Equation (1).

The secrecy capacity is the channel capacity difference between the main channel and eavesdropper channel. From Equation (10), we find that when the relay number M increases, the capacity obtained by each relay decreases because of the reduction of the amplification coefficient. However, the large number of relays can improve the secrecy capacity. Thus, there exists a certain relay number M that maximizes the secrecy capacity. Meanwhile, the position of the relay affects the main channel condition. To obtain the maximum secrecy capacity, we proposed a coalition formation algorithm to select the relays based on coalition game theory because of the distribution property.

## 3. Coalitional Games

### 3.1. Coalition Game Model

In wireless networks, the coalitional game is an effective mathematical tool to enhance the PHY security and improve the performance. As is defined, a coalitional game with transferable utility (TU) is represented by a pair (N,V), where N is the set of players and the function V is a value for each coalition. Denote S as an element in N and ϕ(S) as the payoff of coalition S during its time slot, and the function V is defined as follows [15]:

(14) $$V\left(S\right)=\left\{\phi \left(S\right)\in R^{\left|S\right|},\forall i\in S,\phi \left(S\right)={\left(C^{S}-C_{E}^{S}\right)}^{+}\right\}$$

where $C^{S}$ is the gain in terms of secrecy capacity for coalition S given by (10), $C_{E}^{S}$ is a security loss function for coalition S during the first slot, and the security loss caused by the fth (f∈Z) eavesdropper in the first slot is given by Equation (12). Let $C\left(S\right)={\left(C^{S}-C_{E}^{S}\right)}^{+}$, and then C(S) is the secrecy capacity and acts as the measure of the wireless communication security. The cost function $C_{E}^{S}$ is computed according to Equation (13), which is the security loss caused by eavesdroppers.

### 3.2. Coalition Formation

The coalitional game can be divided into two kinds of orders. One is coalition value orders, and the other is individual value orders. Coalition value orders compare the value of two collections, such as the utilitarian order, in which W⊳Z implies $\sum_{i=1}^{x}v\left(S_{i}\right)>\sum_{i=1}^{y}v\left(S_{i}^{*}\right)$. In contrast, the individual value orders compare the individual payoff of each player, such as the Pareto order. Assuming that the collection W and the collection Z have the same players and the payoff of a player $R_{j}$ in these two collections are denoted by $\phi_{j}^{v}\left(W\right)$ and $\phi_{j}^{v}\left(Z\right)$, respectively, which can be written as $W⊳Z\Leftrightarrow \left\{\phi_{j}^{v}\left(W\right)\geq \phi_{j}^{v}\left(Z\right),\forall R_{j}\in W,Z\right\}$ by Pareto order if the collection W is better than the collection Z. In other words, W is preferred to Z if at least one player’s utility is increased without decreasing other players’ utilities.

In this CCS-AF network, only the coalition that obtains the highest secrecy capacity can be selected, which means only the relays in the coalition with higher secrecy capacity have more chances for cooperation. If a relay cannot participate the coalition with the highest secrecy capacity, it will obtain nothing. Differently from the cases where the individual values are considered, we consider the whole coalition value, where the Pareto order is not suitable. The two coalitions are preferred to be merged into one coalition if a higher coalition value can be obtained. This merge operation makes the players in these two coalitions have more chance to win the utility. To deal with the cooperative problem, a new comparison relation rule is defined as follows.

**Definition** **1.** Consider two collections, $W=\left\{S_{1},\ldots ,S_{l}\right\}$ and $Z=\left\{S_{1}^{*},\ldots ,S_{k}^{*}\right\}$, which have the same players. Then, the ‘Max-Coalition order’ is defined as $W≻Z\Leftrightarrow \left\{max\left\{v\left(S_{1}\right),\ldots ,v\left(S_{t}\right)\right\}\geq max\left\{v\left(S_{1}^{*}\right),\ldots ,v\left(S_{h}^{*}\right)\right\}\right\}$.

### 3.3. Merge-and-Split Rule

To make sure the relays can self-organize to form the coalition, the “merge” and “split” rules are defined as below.

*Merge Rule*: Merge any set of coalitions $\left\{S_{1},\ldots ,S_{l}\right\}$ whenever the relays prefer the merged form.

(15) $$if\;\left\{{⋃}_{j=1}^{l}S_{j}\right\}≻\left\{S_{1},\ldots ,S_{l}\right\},\;then\;\left\{S_{1},\ldots ,S_{l}\right\}\to \left\{{⋃}_{j=1}^{l}S_{j}\right\}$$

*Split Rule*: Split any coalition $\overset{l}{\underset{j=1}{\cup }}S_{j}$ whenever the relays prefer a split form.

(16) $$if\;\left\{S_{1},\ldots ,S_{l}\;\right\}≻\left\{{⋃}_{j=1}^{l}S_{j}\right\},\;then\;\left\{{⋃}_{j=1}^{l}S_{j}\right\}\to \left\{S_{1},\ldots ,S_{l}\right\}$$

Based on the merge-and-split rules and the ‘Max-Coalition order’ proposed above, the relays will form a new coalition if split-and-merge operations improve the secrecy capacity; otherwise, they keep the original coalition.

## 4. Coalition Formation Algorithm

In this CCS-AF network, some of the relays could form a coalition to maximize the system secrecy capacity based on the newly defined Max-Coalition order. Coalitions would like to merge if the merging forms a preferred coalition. It is implied that a group of relays will sign an agreement to merge into a larger coalition if the coalition yielded by the merge has larger secrecy capacity. Similarly, a coalition should split if the splitting can form a preferred coalition. At the beginning, relays are noncooperative nodes and the coalition structure is $W=\left\{\left\{R_{1}\},\ldots ,\left\{R_{k}\right\},\ldots ,\{R_{M}\right\}\right\}$. Therefore, the first operation should be merging. Based on merge-and-split rules and ‘Max-Coalition order’, the coalition formation algorithm is shown in Table 1. The coalition formation algorithm needs to know the transmission matrix to calculate the secrecy capacity of the possible coalition and decide whether to form this possible coalition. This requires that the CSI must be accurate. If the CSI is wrong, the relays cannot obtain the accurate transmission matrix. This makes the relay node unable to calculate the security capacity of the possible coalition correctly. If the CSI error is too large, the new formed coalition based on the ‘Max-coalition order’ may have a lower secrecy capacity than the old coalition. This will make the network unable to select the best relay to obtain the highest security performance through the proposed algorithm.

In order to make the topology adaptive to the relays’ dynamic location, the coalition formation algorithm is repeated periodically during the network operation. There are three stages in the algorithm: neighbor discovery, secrecy capacity calculation, and the iterative coalition formation. After these three stages, the topology is stable and the appropriate coalition will be selected.

In the neighbor discovery stage (stage 1), each coalition surveys the environment and finds the possible neighbor coalition to cooperate with. In the secrecy capacity calculation stage (stage 2), the coalition calculates the secrecy capacity periodically. Then, the iterative formation process begins (stage 3). In this stage, relays determine whether to merge with other coalitions to form a new coalition or sign a split agreement to break the current coalition. If the merge operation can yield a new coalition $S_{k}^{\prime }$ which has a larger secrecy capacity than coalitions $S_{k}$ and $S_{t}$, coalitions $S_{k}$ and $S_{t}$ in $Z_{n}$ will merge based on the Max–Coalition order: the two coalitions $S_{k}$ and $S_{t}$ will form a new coalition, $S_{k}^{\prime }$. This is mainly because merging brings a long-term secrecy capacity improvement of this network. The merging operation will stop if there is not a better merge form. After merging, the coalitions are about to split based on the split rule. If coalitions can find a better split form through the Max-Coalition order, the relays in those coalitions will sign the split agreement to form a new coalition, $S_{k}^{\prime }$. This is because splitting improves the secrecy capacity of the network. The network will repeat the merge-and-split operations until the highest secrecy capacity has been obtained. The coalition $S_{c}$ which can obtain the highest system secrecy capacity is selected to help sources transmit the information. Under this circumstance, the coalition is stable and relays have no incentive to merge or split to form a new coalition.

The complexity of this algorithm is related to the split/merge operations. In terms of the split operation, supposing the current coalition is $S_{k}$, the number of potential coalitions grows exponentially with the number of relays in coalition $S_{k}$. Supposing the number of relays in the current coalition is *M* and the total number of relays in the network is $M_{A}$, the number of merge trails is $\sum_{l=1}^{M_{A}-M}l$ $=\left(M_{A}-M\right)\left(M_{A}-M+1\right)/2$, and this results in the complexity of $o\left({\left(M_{A}-M\right)}^{2}\right)$, while the real computation load is usually far below this, for the algorithm will stop when a better coalition is found. The complexity is the number of all possible merge operations under the current coalition structure, while the real computational load is the number of real operations before the merge operation stops, which means there is no other better coalition with higher secrecy capacity that can be obtained through the merge operation. Obviously, the number of real merge operations is far below the number of all possible merge operations under the current coalition structure. In other words, there is no need for the algorithm to traverse all possible situations, because the iteration of operation will stop once a better coalition is found.

## 5. Simulations

**Figure 2:**
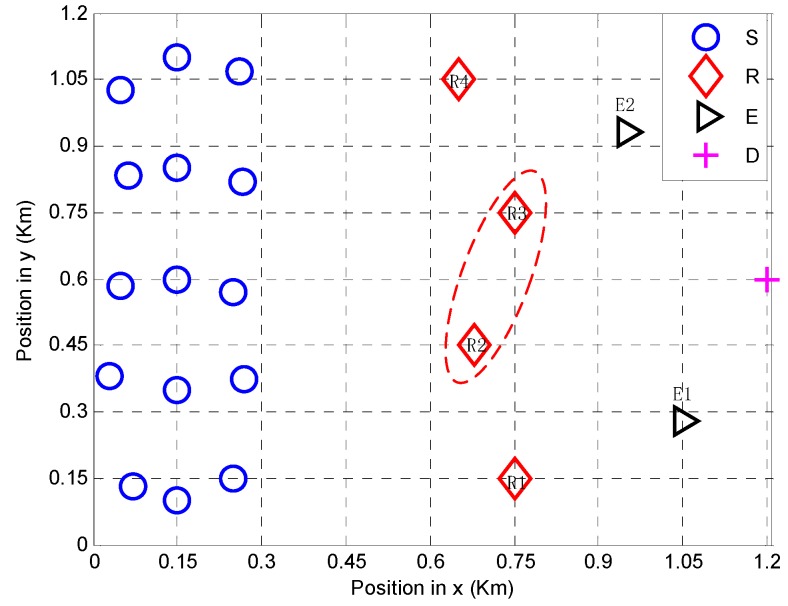
A coalitional structure with eavesdroppers.

We consider a coalitional network with 15 users, four relays, two eavesdroppers, and one destination located in a square of 1.2 km×1.2 km, as shown in Figure 2. Sources are randomly distributed between x=0 to x=0.3. The coordinates for the destination and eavesdroppers are set to (1.2 km, 0.6 km) for D, (1.05 km, 0.28 km) for E1, and (0.95 km, 0.93 km) for E2. The path loss μ is 4, the power constraints of sources and relays are $P_{S}=0\;\mathrm{dBm}$ and $P_{R}=0\;\mathrm{dBm},$ respectively, and both $\sigma_{n_{0}}^{2}$ and $\sigma_{w_{0}}^{2}$ are $10^{-14}\;W$.

### 5.1. Coalition Formation Process

As shown in Figure 2, the coordinates for relays are set to (0.75 km, 0.15 km) for Relay 1 (R1), (0.68 km,0.45 km) for Relay 2 (R2), (0.75 km, 0.75 km) for Relay 3 (R3), and (0.65 km, 1.05 km) for Relay 4 (R4). It is shown in Figure 2 that R3 and R4 form a coalition and help sources to forward the information. The capacities of all potential coalitions and the relay utility are shown in Table 2. The details of the coalition formation process are described below.

At first, for both Max-Coalition order and Pareto order, the relay nodes are noncooperative and each relay is a coalition. The first operation is a merge operation, and it starts with R3 (the nearest relay from the destination), which attempts to merge with R4 (the closest relay to R3). Then, the coalition {R3,R4} is formed. Subsequently, the coalition {R3,R4} tries to merge with R2 (the nearest relay to the coalition {R3,R4}) to form a three-relay coalition. Then, the process of Max-Coalition order and Pareto order will be described separately.

For Max-Coalition order, the coalition {R3,R4,R2} is formed successfully because the system secrecy capacity is improved from 1.4087 to 1.4583. After that, the coalition {R3,R4,R2} has an incentive to merge with R1, but this merge is impossible as the system utility decreases (1.2593<1.4583). Until this moment, the merge operation is finished, for no other better coalition can be obtained by the merge operation, and the split operation begins. For the first split operation, R4 (located in the edge of the current coalition) splits from the coalition {R3,R4,R2} successfully, because the split operation can enhance the secrecy capacity from 1.4583 to 1.5547. Then, the iteration process ends. Thus, the coalition {R3,R2} is the selected coalition, and its utility is v({R3,R2})=1.5547. As a result, for Max-Coalition order, the coalition {R3,R2} cannot merge or split any further, and the final coalition structure is W={{R3,R2},{R1},{R4}}.

In contrast, based on Pareto order, the coalition {R3,R4,R2} cannot be formed. This is because the utilities of R3 (0.6257<1.0481) and R4 (0.1701<0.3606) cannot be improved through this merge. Then, the coalition {R3,R4} tries to merge with R2, but the merge operation fails for the same reason (for R3 0.5402<0.6257 and for R4 0.1522<0.3606). For Pareto order, the coalition {R3,R4} is selected and its utility is v({R3,R4})=1.4087. It can be seen that the utility obtained by Max-Coalition order is higher than that of the Pareto order, and this result demonstrates the effectiveness of the Max-Coalition order.

### 5.2. The Adaption on the Relay’s Mobility

**Figure 3:**
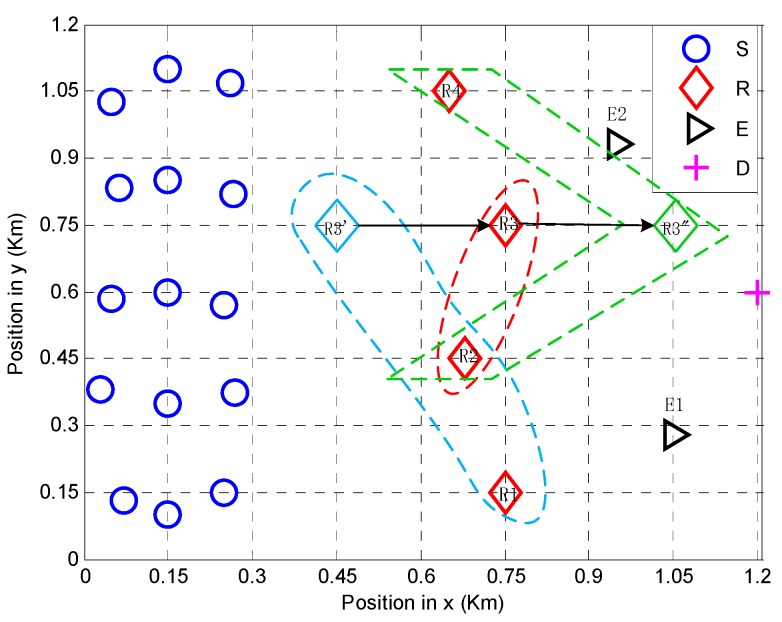
Coalition structure with the movement of *R*3.

**Figure 4:**
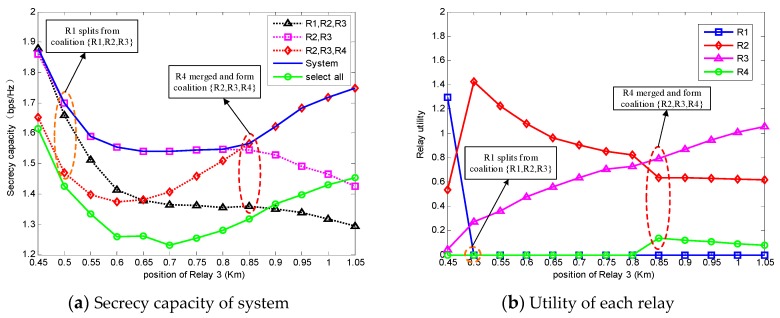
System secrecy capacity and the relay utilities with the movement of Relay 3 corresponding to Figure 3.

In this section, the algorithm’s ability for handling the relay’s mobility is studied. For this purpose, R3 in Figure 2 is moved horizontally from 0.45 km  (R3′) to 1.05 km  (R3″). The change of the coalition structure and the performance are shown in Figure 3 and Figure 4, respectively.

In Figure 3, the coalition structure changes dynamically when relay R3 changes its location. When relay R3 moves from sources to the destination, R1 first splits from {R1,R2,R3}. When R3 moves close to destination, R4 merges and forms the coalition {R2,R3,R4}.

Figure 4a is the secrecy capacity of the system, and Figure 4b is the utility of each relay accordingly. In Figure 4a, it is obvious that the system secrecy capacity obtained by the coalition formation algorithm is much higher than the circumstance when all relays are selected. This phenomenon proves that the coalitional game algorithm improves the system security.

Obviously, the selected coalition through the coalition formation algorithm is stable based on the Max-Coalition order, because there is no relay that has an incentive to split or merge and form another coalition which can obtain a higher system secrecy capacity. Due to the fact that the algorithm prefers a higher secrecy capacity to keep the stability of the coalition, the coalition structure will change to obtain a higher capacity when R3 moves from 0.45 km  to 1.05 km . We can easily know that the splitting of R1 at 0.5 km  is beneficial to both R2 and R3 from Figure 4b, so R1 splits from {R1,R2,R3}. During the movement of R3, the utility of R3 is increasing, and this is because R3 is getting closer to the destination. After R3 moves at 0.85 km , the system capacity starts increasing. It is clear that the merging of R4 increases the utility of R3 and the capacity of the system.

Obviously, the secrecy capacity obtained through the proposed algorithm is higher than the secrecy capacity obtained with all relays. This is because the relays can self-organize into some coalitions, and the best coalition structure will be formed after some iteration guided by the ‘Max-Coalition order’. Due to the property of the ‘Max-Coalition order’, the relays will try to form the coalitions which can obtain a higher secrecy capacity. Thus, the final formed coalition structure is the best structure and it is stable because relays cannot form another structure with a higher secrecy capacity. In the final state, the relays in the best coalition which obtains the highest system secrecy capacity will be selected to transmit the source information. Therefore, we can easily know that the selected relays have a higher ability to obtain the secrecy capacity than the other unselected relays, and the total relay power will be only allocated to these selected relays. In other words, this relay-selection algorithm can select better relays and increase the efficiency of the total relay power. Thus, the secrecy capacity is improved by the proposed algorithm.

### 5.3. The Effect of Power on the Performance

**Figure 5:**
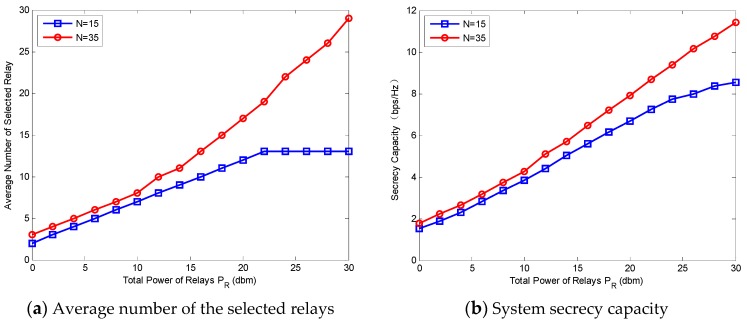
System performance and the average size of coalition versus $\mathrm{power}\;\mathrm{of}\;\mathrm{relay}\;P_{R}$.

Figure 5 shows the system performance and the average size of coalitions versus $P_{R}$, where the number of sources N  is set to 15 and 35, respectively. The total power of sources $P_{S}=0\;\mathrm{dBm}$ and $P_{R}$ varies from 0 dBm to 30 dBm. Figure 5a is the average number of the selected relays with different $P_{R}$. It can be observed that the average number of selected relays increases with the increase of $P_{R}$. This implies that the higher $P_{R}$ is, the more relays that have a chance to gain their utilities. Compared with the circumstance when N=15, especially when $P_{R}$ is large, the number of selected relays when N=35 is higher. When $P_{R}\geq 22\;\mathrm{dBm}$, the number of selected relays (when N=15) is no more than 15; this can be explained by Equation (2). Figure 5b demonstrates the maximum system secrecy capacity when the number of selected relays is set as in Figure 5a. The secrecy capacity with each N increases with the increase of $P_{R}$. In other words, higher $P_{R}$ results in a better system performance.

**Figure 6:**
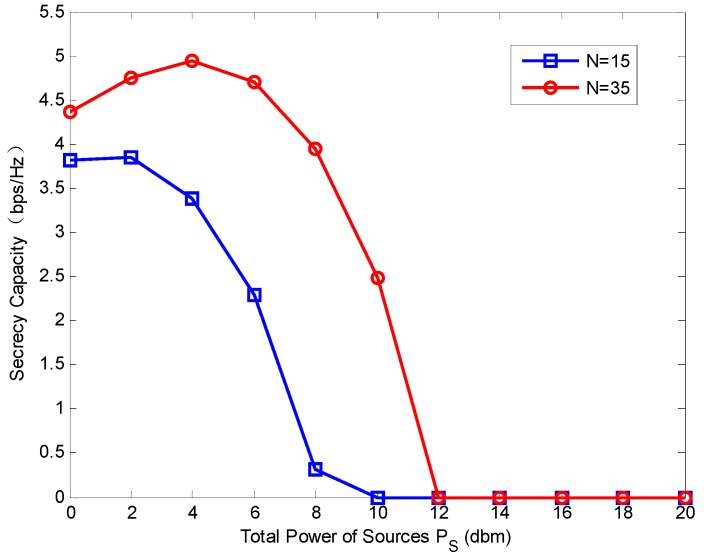
System capacity with different number of sources versus $P_{S}$.

**Figure 7:**
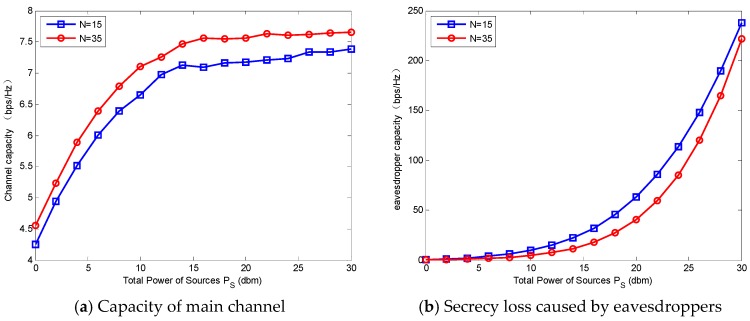
Channel capacity and eavesdropper capacity with different number of sources versus $P_{S}$.

Figure 6 represents the secrecy capacity versus $P_{S}$ where the number of sources N  is set to 15 and 35, respectively. The total power of relays $P_{R}=0\;\mathrm{dBm}$ and $P_{S}$ varies from 0 dBm to 18 dBm. The secrecy capacity is first increased and then decreased along with the increasing of the $P_{S}$. When $P_{S}$ is larger than a certain value, the secrecy capacity will drop to zero (for N=15 and 35, the certain values of $P_{S}$ are 10 dBm  and 12 dBm, respectively). This means the too-large $P_{S}$ has a negative effect on the system security. This phenomenon can be explained by Figure 7a,b. When $P_{S}$ increases, the channel capacity goes up first and then stays steady at the point about $P_{S}=15\;\mathrm{dBm}$. While the secrecy loss caused by eavesdroppers increases slowly at first, and at the point about $P_{S}=15\;\mathrm{dBm}$, it goes up rapidly. In other words, when $P_{S}$ is larger than a certain value, the increase of eavesdropper capacity is much faster than that of the channel capacity. Thus, the large $P_{S}$ degrades the secrecy capacity.

## 6. Conclusions

In this paper, a distributed multi-relay selection algorithm based on the coalition formation game is proposed to enhance the physical layer security of the CCS-AF wireless network, which consists of multiple sources and multiple relays with compressed sensing. Compared with the traditional ‘Pareto order’, the newly defined ‘Max-Coalition order’ rule is proved to be more suitable to solve the multi-relay selection problem for the CCS-AF networks. Simulation results show that the relays can self-organize into a better coalition and have good ability to adapt to the change of the topology. The proposed algorithm can select the better relay nodes and increase the efficiency of the total relay power. If the CSI is accurate, the system security performance can be improved significantly through the distributed relay selection algorithm.

## Figures and Tables

**Table 1:** Coalition formation algorithm.

**Initial State:** The coalition structure of the network is $W=\left\{S_{1},\ldots ,S_{k}\right\}$ (at the beginning, all relays are noncooperative nodes, i.e., $W=\left\{\left\{R_{1}\},\ldots ,\left\{R_{k}\right\},\ldots ,\{R_{M}\right\}\right\}$). **Stage 1—Neighbor discovery** Each coalition surveys the environment and finds the possible neighbor to cooperate with. **Stage 2—Secrecy capacity calculation** Calculate the secrecy capacity V of the coalition $S_{k}$. **Stage 3—Iterative coalition formation** In this phase, a stable coalition structure is formed to obtain a higher secrecy capacity by using merge-and-split rules. The number of selected relays is limited in that $2K\leq M_{c}<N$. **Repeat** (a) $Z_{n}=$ **Merge** ($W_{n}$): some coalitions in $W_{n}$ decide to merge with each other based on the Max-Coalition order, if the relay number of the new coalition is less than the source number, i.e., $M_{c}<N$. If the merge can yield a new coalition $S_{k}^{\prime }$ which has a larger secrecy capacity than the coalitions $S_{k}$ and $S_{t}$, coalitions $S_{k}$ and $S_{t}$ will merge to form the new coalition $S_{k}^{\prime }$. Otherwise, relays stay in the former coalition; coalitions $S_{k}$ and $S_{t}$ do not change. (b) $Z_{n}=$ **Split** ($W_{n}$): the coalition in this network decides to split based on Max-Coalition order, if the relay number of the new coalition is two times larger than the sparsity, i.e., $M_{c}>2K$. (c) n=n+1. **Until merge-and-split terminates.** **Final state: Coalition selected** The coalition $S_{c}$ which can obtain the highest system secrecy capacity is selected to transmit the information. **Stage 1 to Stage 3 are repeated periodically when the network operates, and this repetition makes sure the network topology is adaptive to the change of environment (such as mobility of relays).**

**Table 2:** The utility of each potential coalition and each relay for the network of Figure 2.

**Cooperative with All Relays**											
v({R1,R2,R3,R4})=1.2539, v({R1})=0.4990, v({R2})=0.3410, v({R3})=0.3410, v({R4})=0.0751											
**Cooperative Using Coalition Formation Algorithm**											
**Max-Coalition order**						**Pareto order**					
	system	*R*1	*R*2	*R*3	*R*4		system	*R*1	*R*2	*R*3	*R*4
{R3,R4}	1.4087	-	-	1.0481	0.3606	{R3,R4}	1.4087	-	-	1.0481	0.3606
{R3,R4,R2}	1.4583	-	0.6625	0.6257	0.1701	{R3,R4,R2}	1.4583	-	0.6625	0.6257	0.1701
{R3,R4,R2,R1}	1.2539	0.4990	0.3410	0.3388	0.0751	{R3,R4,R1}	1.2906	0.5982	-	0.5402	0.1522
{R3,R2}	1.5547	-	0.8514	0.7033	-						
